# Determination of the Faraday rotation perpendicular to the optical axis in uniaxial CeF_3_ crystal by using the Generalized-High Accuracy Universal Polarimeter

**DOI:** 10.1038/s41598-019-54174-2

**Published:** 2019-12-05

**Authors:** Kenta Nakagawa, Toru Asahi

**Affiliations:** 1 Kanagawa Institute of Industrial Science and Technology (KISTEC), Kanagawa, 243-0435 Japan; 2 Graduate School of Advanced Science and Engineering, Department of Advanced Science and Engineering, Waseda University, Tokyo, 162-8480 Japan; 3 Global Consolidated Research Institute for Science Wisdom, Waseda University, Tokyo, 162-0041 Japan

**Keywords:** Materials for optics, Optical materials and structures

## Abstract

Many single crystals have been developed and commercialized for optical isolators. However, optical isolator materials have been limited to isotropic crystals or to the isotropic direction (optic axis) of anisotropic crystals. This study investigates the wavelength dependences of linear birefringence, linear dichroism, Faraday rotation and magnetic-circular dichroism in a single crystal rare-earth fluoride, namely CeF_3_. Measurements were made in the direction parallel and perpendicular to the optic axis under an applied magnetic field. The magnetic field was generated by Nd-Fe-B magnets installed in the generalized-high accuracy universal polarimeter (G-HAUP). The first application of G-HAUP to a magneto-optical material is presented. In the CeF_3_ crystal, the Verdet constants along directions parallel and perpendicular to the optic axis were positive over the measured wavelength region (300–680 nm), and their magnitudes were nearly equal. The success in the accurate measurement on Faraday rotation along anisotropic directions has opened the way to study on optical isolators along the direction other than optic axis.

## Introduction

Avoiding parasitic oscillations and frequency instabilities in amplifier systems is imperative in optical communication support for advanced information technology and Internet society. Frequency instabilities, which decrease the quality, stability and lifetime of a laser light source, have received much attention^1,2^. Optical isolators are based on the Faraday rotation (FR), which is a non-reciprocal magneto-optical rotation of polarization of light. FR is explained using the imaginary and anti-symmetrical part of the dielectric tensor induced by an applied magnetic field *H*^3^ in a medium. FR is generally expressed as an odd function of *H*^4^ but for sufficiently weak *H* the FR angle can be approximately designated by the following equation^5,6^:

1 $$\theta =VHL,$$

where *V* and *L* are the Verdet constant and the sample thickness, respectively.

Numerous single crystals have been developed and a few have been commercialized for optical isolators^7–14^. However, the materials of optical isolators have been limited to isotropic crystals or to the isotropic direction, (*i*.*e*., along the optic axis) of anisotropic crystals. Optically anisotropic crystals are disadvantaged by linear birefringence (LB) and linear dichroism (LD), since a light beam splits into two orthogonally linear-polarized components with different refractive indices and absorption coefficients, respectively. The differences can be two to four orders of magnitude higher than the refractive-index difference between the right and left circularly-polarized light components in circular birefringence^15,16^. In other words, FR in anisotropic crystals cannot be accurately measured by conventional optical apparatuses such as polarimeters and circular dichroism (CD) spectrophotometers, except along special directions such as the optic axis. Therefore, optically anisotropic directions in anisotropic crystals have been almost ignored in the magneto-optical research field.

Nevertheless, the influence of LB on FR in an anisotropic medium has attracted some interest. Chauvin measured the Verdet constants of calcite along directions slightly inclined from the optic axis, but no dependence of these constants on the light propagation direction was observed^17^. Ramaseshan derived an approximate negative correlation between FR and LB, and verified it in experiments on strained glasses and plastics^18^. It should be noted that FR has been ambiguously defined in some previous reports^18,19^. When linearly-polarized light enters an optically anisotropic medium along an external magnetic field, it splits into two orthogonal elliptically-polarized rays of opposite senses, traveling at different velocities. When emitted from the surface of the medium, these two vibrations superimpose to produce an elliptically-polarized light whose major axis is rotated (except in special cases) from the plane of the linearly-polarized incident light. This rotation angle, called the *apparent rotation* in some reports, was defined as the FR in Ramaseshan. We considered that the definition of *apparent rotation* is not equal to that of Faraday angle. As the superimposed polarized light become elliptical and the major axis rotates when the LB and LD exist, it is difficult to estimate whether or how FR contributes to the rotation. Later, Tabor *et al*. analyzed light propagation through a medium exhibiting both FR and LB from an electro-magnetic perspective, and suggested ways of measuring these physical quantities^20^. They measured the visible and infrared FR and LB in single crystals of rare-earth orthoferrites using transmission techniques. They also recognized the difficulties of applying a material with LB to optical devices based on FR^21^.

**Figure 1:**
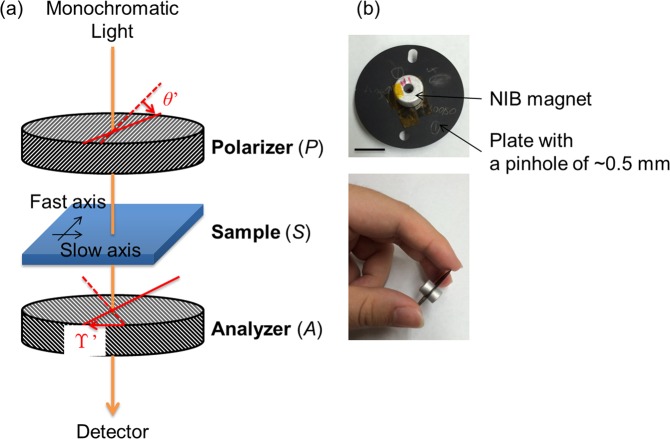
(**a**) Optical configuration of the G-HAUP apparatus. The axes of polarizer (*P*) and analyzer (*A*) are set in the crossed-Nicols configuration. *θ*′ and Υ′ are the azimuthal angles of *P* from the extinction position and *A* from the crossed-Nicols configuration for the extinction position of *P*, respectively. (**b**) Photograph of the NIB magnet to apply the magnetic field parallel or anti-parallel to the light propagation direction. The mounted sample on the plate with a pinhole of diameter ~0.5 mm is sandwiched between two identical ring NIB permanent magnets. The black line represents the scale bar (1 cm).

The subject material selected Faraday rotator in the present study CeF_3_ single crystal, is characterized by its wide transparency range (300 nm to 2500 nm) and an outstanding Verdet constant, besides of being uniaxial^22–24^. Cerium is usually found as a trivalent ion Ce^3+^ in condensed matter. The electronic configuration of Ce^3+^ is 1*s*^2^2*s*^2^2*p*^6^ … 4*d*^10^ 4*f* ^1^5*s*^2^5*p*^6^. Electronic transitions from 4*f* to 5*d* confer the magneto-optical properties observable in the UV-Vis-IR region^1^. In a previous report^23^, the refractive indices of the ordinary and extraordinary light rays in single crystal CeF_3_ at 633 nm were determined as 1.616 and 1.609, respectively. Therefore the LB is 0.007, comparable to that of the *α*-quartz crystal along its *a* axis. Even at this low order of LB magnitude (10^−3^), the OR of the crystal cannot be accurately measured by conventional optical apparatuses^25^. Previously, we developed a novel optical apparatus called the generalized-high accuracy universal polarimeter (G-HAUP), which simultaneously measures the wavelength dependences of the OR, CD, LB, and LD in an anisotropic medium (Fig. 1(a))^26,27^. When a sample is subjected to a magnetic field applied parallel/anti-parallel to the light propagation direction, the G-HAUP can measure its FR and Magnetic-CD (MCD). In this study, we measured the wavelength dependences of the FR, MCD and optical anisotropy in CeF_3_ single crystal along the optic axis (*c* axis) and perpendicular to the optic axis (*a* axis) with the G-HAUP. A magnetic field parallel/anti-parallel to the light propagation was generated by Nd-Fe-B (NIB) magnets introduced for that purpose (Fig. 1(b)).

## Results

**Figure 2:**
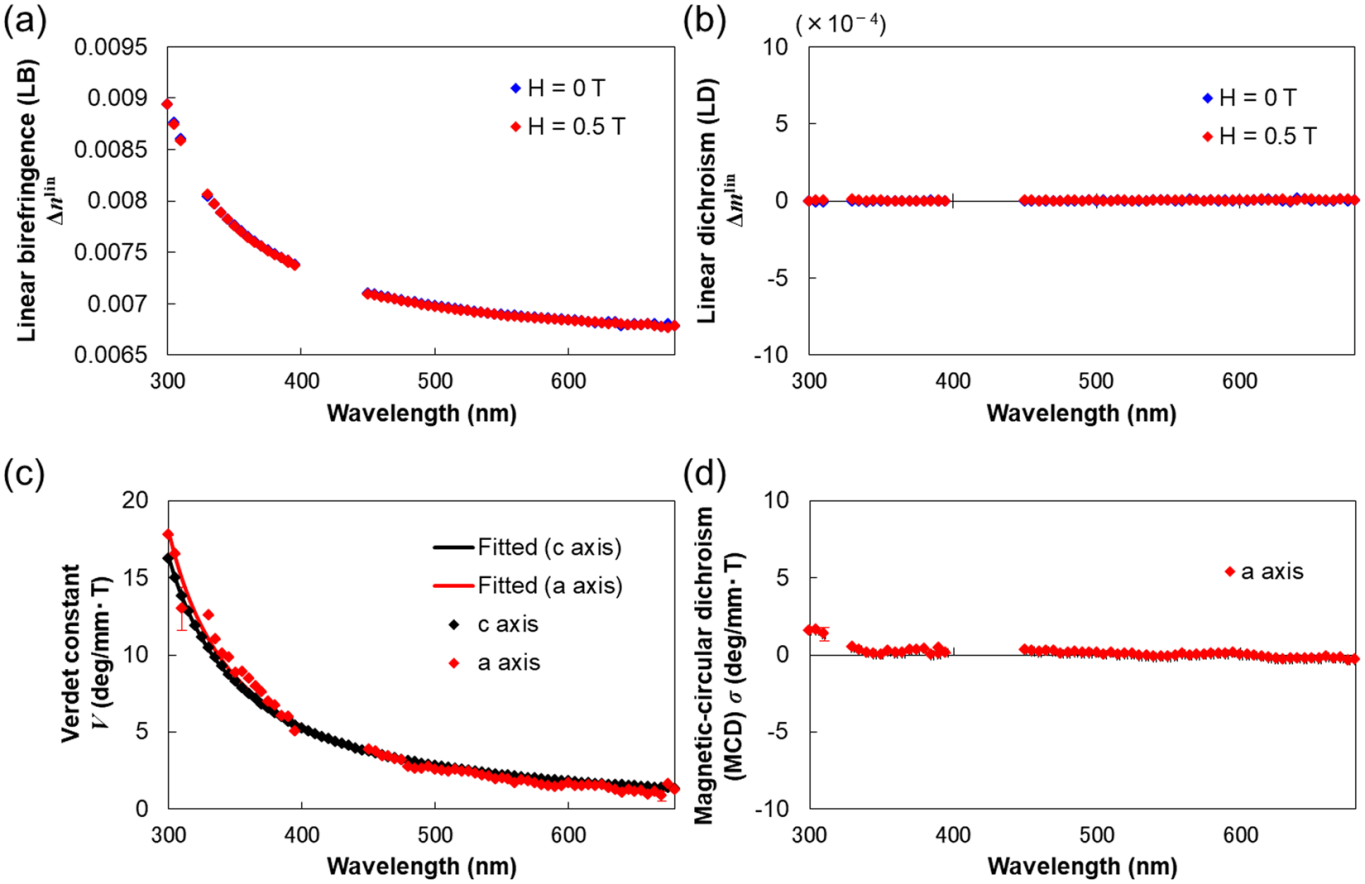
Wavelength dependences of the LB (**a**), LD (**b**), Verdet constant (**c**) and MCD (**d**) in single-crystal CeF_3_ at 25 °C. The black, blue and red rhombuses represent the data along the *c* axis, the *a* axis without a magnetic field and the *a* axis under an applied magnetic field, respectively. We eliminated the anomalous behaviors of Verdet constant and MCD due to the unstable and low-sensitivity regions in the G-HAUP measurements. The Verdet constant spectra were fitted to a simple Drude oscillator. The error bars represent the standard deviations. The error range for *a* axis Verdet constant in (**c**) is 0.32–1.40 deg/mm · T.

We prepared a 307-*μ*m thick (001) plate of single-crystal CeF_3_ by polishing. In structure, the CeF_3_ crystal belongs to the uniaxial and optically inactive crystal point group *D*_3*d*_ (Table S1). Therefore, when the magnetic field is applied parallel to the light propagation direction, FR occurs only along the *c* axis. Although FR along the optic axis is easily measured by conventional optical apparatuses, we here implemented the rotating analyzer mode of G-HAUP^28^. To apply the magnetic field parallel or anti-parallel to the light propagation direction, we mounted the sample on a pinhole plate, and sandwiched it between two ring NIB permanent magnets. The magnetic field in the middle of the magnets was homogeneous, with a value of ~0.5 T parallel to the longitudinal direction^29^. The wavelength dependences of the Verdet constant along the *c* axis at 25 °C are plotted as black rhombuses in Fig. 2(c). The Verdet constants along the *c* axis were positive over the observed wavelength region, indicating that the right-handed circularly-polarized light propagates faster than its left-handed counterpart.

We then prepared a 58.0-*μ*m thick (100) plate sample of single-crystal CeF_3_ by polishing. The optical characters, *i*.*e*., the directions of the fast and slow light rays, were determined under a polarizing microscope (DMLP, Leica, Hesse, Germany) with a Ehringhaus compensator. Before measuring the magneto-optical properties, we determined the wavelength dependences of the LB, LD, OR and CD along the *a* axis in the absence of the magnetic field at 25 °C (blue rhombuses in Fig. 2). For these measurements, the G-HAUP was operated in extended mode^26,27^. (The measurement principles of the G-HAUP and the data analysis of the G-HAUP measurements are described in the Supplementary Information). The LB along this axis was of the same order of magnitude as the LB of *α*-quartz crystal (Fig. 2(a)). However, the LDs were almost zero over the wavelength region (Fig. 2(b)). This result is consistent with the UV-Vis spectrum, which exhibits no significant absorption above 282 nm. As shown in Fig. S2(c,d), the OR and CD values were also close to zero, consistent with the clack of optical activity for this crystal structure.

Then, the wavelength dependences of the LB, LD, FR, and MCD along the *a* axis under a magnetic field parallel to the light propagation direction at 25 °C were measured in the extended mode of G-HAUP (red rhombuses in Fig. 2). In general, reversing the magnetic field direction inverted the signs of FR and MCD. Therefore, in order to obtain accurate spectra, we applied the magnetic field anti-parallel to the light propagation direction, re-measured the wavelength dependences, and averaged the absolute magnitudes of both sets of measurements. The values of LB and LD hardly changed with and without the magnetic field (Fig. 2(a,b)). The Verdet constants along the *a* axis were positive throughout the wavelength region (Fig. 2(c)).

## Discussion

As it is well known, the light propagation in an anisotropic crystal can be described by the Maxwell equations and by a material equation describing the macroscopic properties of the material. In general, optical phenomena are little affected by the magnetic permeability tensor *μ*_*ij*_(*ω*, **k**), so we can equate *μ*_*ij*_(*ω*, **k**) to the magnetic permeability in a vacuum. Furthermore, the dielectric permeability tensor *ε*_*ij*_(*ω*, **k**) needs to take into account the magnetization **M** induced by the magnetic field as:

2 $${\varepsilon }_{ij}(\omega ,{\bf{k}})={\varepsilon }_{ij}^{0}(\omega ,{\bf{k}})+\sum _{k}\,{f}_{ijk}{{\bf{M}}}_{k}+\sum _{k}\,\sum _{l}\,{f}_{ijkl}{{\bf{M}}}_{k}{{\bf{M}}}_{l},$$

where ${\varepsilon }_{ij}^{0}(\omega ,{\bf{k}})$ is the dielectric tensor without the applied magnetic field, and *f*_*ijk*_ and *f*_*ijkl*_ are the third- and fourth-rank axial tensors, respectively^30^. Eremenko and Kharchenko analyzed the relationships between the symmetries and optical properties of nonmagnetic materials. They demonstrated FR and MCD under an applied magnetic field along the light propagation direction, and magnetic LB and magnetic LD under an applied field perpendicular to the light propagation direction^31^. In this study, the wavelength dependences of FR in single-crystal CeF_3_ were successfully measured by the G-HAUP along both the *c* and *a* axes under an applied magnetic field. As the LB and LD values were almost identical in the presence and absence of the magnetic field (Fig. 2(a,b)), we concluded that no magnetic field was applied perpendicular to the light propagation direction.

Spin-orbital coupling splits the ground state of the 4*f* configuration of Ce^3+^ ions into several multiplets, which are further split by the crystal field. Under a magnetic field, the degenerate levels split into sublevels by violation of the time-reversal symmetry (Zeeman splitting). According to previous reports, the FR of CeF_3_ arises from the occupation probabilities of these sublevels. Each sublevels are excited by left or right-circularly-polarized light, respectively^32,33^. The FR of paramagnetic lanthanide ions is quantified by the Verdet constant

3 $$V=\frac{\pi {\nu }^{2}\chi }{2{\mu }_{B}ch}\sum _{ij}\,\frac{{C}_{ij}}{{\nu }_{ij}^{2}-{\nu }^{2}},$$

where *v* is the frequency of the incident light, and *v*_*ij*_ and *C*_*ij*_ denote the frequency and probability of transitions between the electronic states *i* and *j*, respectively, *χ* is the magnetic susceptibility, *g* is the Landé factor, *μ*_*B*_ is the Bohr magneton, *c* is the velocity of light and *h* is the Planck constant.

**Figure 3:**
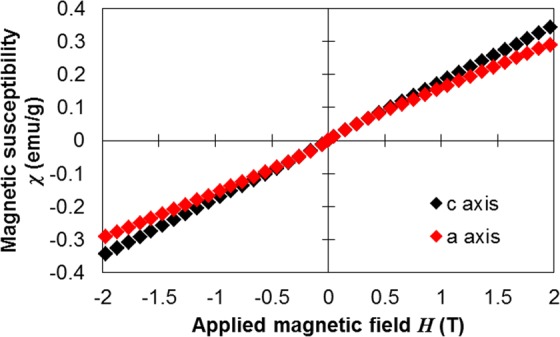
Magnetic susceptibilities of single-crystal CeF_3_ along both the *c* axis (black) and *a* axis (red) at 27 °C.

According to Eq. (3), the magnitude of *V* at an arbitrary frequency *v* depends on the magnetic susceptibility *χ* and the transition probabilities *C*_*ij*_. In this study, the Verdet constants were positive along the *c* and *a* axes at all wavelengths in the measured range, and their magnitudes along both axes were nearly equal (Fig. 2(c)). To investigate these results, Fig. 3 plots the magnetic field dependences of the magnetic susceptibilities of single-crystal CeF_3_ along the *c* and *a* axes at 27 °C. The magnetic susceptibilities, measured with the SQUID instrument (VSM SQUID, Quantum Design, California, USA), were nearly identical along both axes. In addition, the Verdet constants along each the axes, respectively were fitted to a simple oscillator equation, such that

4 $$V=\frac{A}{{\lambda }^{2}-{\lambda }_{0}^{2}},$$

where *A* is a constant appropriate to the absorption wavelength *λ*_0_. The fitted *A* and *λ*_0_ values were 5.3516 × 10^5^ and 239.23 nm along the *c* axis and 5.3553 × 10^5^ and 245.42 nm along the *a* axis, respectively, and the coefficient of determination, *R*^2^, obtained by the fitting procedures were 0.999 and 0.986, respectively. The values of *A* and *λ*_0_ along the *a* axis are almost as large as the values along the *c* axis. In the measured wavelength region, 4*f*–5*d* electronic transitions are associated with the optical and magneto-optical properties of single-crystal CeF_3_. Therefore, we may reasonably consider that the anisotropy of the summed 4*f*–5*d* transition probabilities *C*_4*f*−5*d*_ is zero.

## Conclusion

The wavelength dependences of LB, LD, FR and MCD were investigated along the *a* and *c* axes of single crystal CeF_3_ under an applied magnetic field. These measurements were successfully collected by the G-HAUP equipped with NIB magnets. Before this study, the G-HAUP had never been applied to a magneto-optical material. The Verdet constants along the *c* and *a* axes were positive and nearly equal in magnitude in the measured wavelength region. The results suggest that a novel optical device, based on FR along axes other than the optic axis may be fabricated. When linearly-polarized light enters an optically anisotropic medium, it divides into two orthogonally linearly-polarized components with different refractive indices and absorption coefficients. Therefore, to fabricate an apparently optically isotropic device, we can set two single crystals of the same-thickness under a polarizing microscope, to compensate for the LB and LD. Although, the optical device consists of two optically anisotropic crystals, it should exhibit only FR. As a next step, we should produce and test the performance of such optical isolator. Furthermore, if the FR of an anisotropic crystal is higher in the perpendicular direction than in the parallel direction to the optic axis, it is possible to fabricate a novel optical isolator that operates under much lower magnetic fields. This in turn enables the use of significantly smaller magnets, which favors both miniaturization and cost-down.

## Methods

### Single-crystal growth

Single crystals of CeF_3_ were grown by the modified Czochralski technique are described elsewhere^22,23,34^ and were provided by Prof. Dr. Kiyoshi Shimamura (National Institute for Materials Science, Japan).

### Introduction of NIB magnets to the G-HAUP

In the G-HAUP optical system, the sample was mounted on a Cu plate pinhole (diameter ~0.5 mm) to improve the signal-to-noise ratio of the transmitted light intensity. To apply the magnetic field parallel or anti-parallel to the light propagation direction, we sandwiched the mounted sample between two identical ring NIB permanent magnets (Fig. 1(b)). The outer diameter, inner diameter and length of each magnet were 9 mm, 3 mm, and 5.5 mm, respectively. The middle of the magnet exhibited an homogeneous magnetic field of ~0.5T parallel to the longitudinal direction^29^.

### G-HAUP measurements

G-HAUP employs a simple optical configuration that contains only two optical elements: polarizer (*P*) and analyzer (*A*) (Fig. 1(a)). The axes of the *P* and *A* are set in the crossed-Nichols configuration, and light travels through the *P*, the sample (*S*), and the *A* successively. The G-HAUP is operated by measuring the transmitted light intensity as a function of rotations of the *P* and *A* at discrete azimuthal orientations, from which LB, LD, OR (ORP or FR) and CD can be extracted from the functional dependence of the intensity. Systematic errors; parasitic ellipticities *p* and *q* originating from imperfection of the *P* and *A*, respectively, and a small error angle *δ*Υ attributed to the deviation from the correct crossed-Nicols configuration, are considered into the measuring theory of the G-HAUP and can be removed through the post-experimental procedures to obtain the accurate values of OR and CD. Measurement principle of the G-HAUP is only applied for crystal in anisotropic direction. In the case where measurement of Verdet constant in isotropic direction, Verdet constant is easily measured by conventional optical apparatuses such as polarimeter, but here it was measured by just rotating the analyzer of the G-HAUP.

In this study, we decided to follow the sign convention that is mainly accepted in the field of chemistry. The observer views from the detector looking towards the light source. When the plane of a polarization rotates clockwise, *i*.*e*., *θ* > 0, it is said to be *dextrorotatory*, whereas when the plane of a polarization rotates counterclockwise, *i*.*e*., *θ* < 0, it is said to be *levorotatory*.

We prepared two types of samples, (001) and (100) plates of single-crystal CeF_3_. The sample surfaces were polished by polishing machines (Nano factor, Tokyo, Japan and Metaserv 2000, Buehler, Illinoi, USA) with a diamond slurry (abrasive grain diameter 3.0 *μ*m) and colloidal silica (32.5 nm), yielding thin samples with smooth flat surfaces.

### Magnetic susceptibility measurements

The applied magnetic field dependences of the magnetic susceptibilities of single-crystal CeF_3_ along the *c* and *a* axes at 27 °C were measured with SQUID (VSM SQUID, Quantum Design, California, USA).

## Supplementary information

Supplementary information
