# Supplementary material for: Determination of the Faraday rotation perpendicular to the optical axis in uniaxial CeF3 crystal by using the Generalized-High Accuracy Universal Polarimeter
Source: Sci Rep. 2019 Dec 5;9:18453. doi: 10.1038/s41598-019-54174-2 (PMC6895080; doi:10.1038/s41598-019-54174-2)
Supplement: Supplementary file 1 — Supplementary information [file 41598_2019_54174_MOESM1_ESM.pdf]

Supporting information for: Determination of the Faraday rotation perpendicular to the optical axis in uniaxial CeF_3 crystal by using the Generalized-High Accuracy Universal Polarimeter

Kenta Nakagawa[†,‡,¶] and Toru Asahi[*,‡,¶]

†*Kanagawa Institute of Industrial Science and Technology (KISTEC), Kanagawa, 243-0435, Japan*

‡*Graduate School of Advanced Science and Engineering, Department of Advanced Science and Engineering, Waseda University, Tokyo, 162-8480, Japan*

¶*Global Consolidated Research Institute for Science Wisdom, Waseda University, Tokyo 162-0041, Japan*

E-mail: tasahi@waseda.jp

Single-crystal X-ray diffraction analysis

Single-crystal X-ray diffraction analysis of the grown single-crystal was carried out in a laboratory-scale single-crystal X-ray diffractometer (R-AXIS RAPID-F, Rigaku, Tokyo, Japan), with a graphite monochromated MoK$_\alpha$ radiation (λ = 0.71075 Å) at -100 °C. The structure was solved by direct methods (SIR92)[S1] and expanded using Fourier techniques. All atoms were refined anisotropically. The calculations were performed in the crystallographic software package CrystalStructure 4.2 (Rigaku, Tokyo, Japan), except for the refinement, which was computed in the SHELXL program (Version 2014/6.)[S2]

The crystal data, structure solution, and refinement details of the single-crystal CeF_3 are summarized in Table S1. The crystal system, space group, and lattice parameters of the crystal agree with previously reported findings.[S3] The crystal structure views along the c and a axes are illustrated in Figure S1. The coordination sphere of the Ce^{3+} ion contains 11 F^- ions. The Ce^{3+} ion is almost centralized in the distorted triangular prism formed by 6 F^- ions. The 3 F^- ions on the straight line connecting the Ce^{3+} ion and the center of each side surface of the distorted triangular prism are located in the ab plane. The 2 F^- ions on the straight line connecting the Ce^{3+} ion and the center of the bottom surfaces align along the c axis.

Measurement principles of the G-HAUP

Here, we briefly explain the principles of the HAUP methods. The same optical systems are used in the original HAUP[S4] and the extended HAUP methods[S5,S6] are same. The HAUP methods employ a simple optical configuration with only two optical elements: a polarizer (P) and an analyzer (A). The axes of P and A are set in the crossed-Nicols configuration, and a light ray successively travels through P, the sample (S), and A.

In this simple optical configuration, systematic errors, originating from parasitic ellipticities of P and A (p and q, respectively) and the small error angle attributed to the

displacement of the crossed-Nicols configuration ($\delta\Upsilon$) are evaluated and eliminated.[S7] Here, θ and Υ are the azimuthal angles of P from an arbitrary origin and A from the crossed-Nicols configuration of the arbitrary origin of P's arbitrary origin, respectively. θ_0 is the extinction position angle (*i.e.*, $(\frac{\delta(I/I_0)}{\delta\theta})_{\Upsilon'=0} = 0$) of P from its arbitrary origin, and θ' and Υ' are the azimuthal angles of P from θ_0 and A from Υ', respectively. That is, $\theta = \theta_0 + \theta'$ and $\Upsilon = \delta\Upsilon + \Upsilon'$.

In the extended HAUP method, the relative intensity ratio Γ of the light transmitted from the HAUP simple optical configuration is given as

$$\Gamma = I/I_0 = |\mathbf{A^T M_H P}|^\mathbf{2} \tag{1}$$

where I and I_0 represent the intensities of the transmitted and incident light, respectively, in the HAUP configuration, $\mathbf{P}$ and $\mathbf{A}$ represent the polarization states of P and A, respectively, and $\mathbf{M_H}$ represents the Jones matrix of S.

The Jones vectors $\mathbf{P}$ and $\mathbf{A}$ are given by

$$\mathbf{P} = \begin{pmatrix} \cos\theta\cos p + i\sin\theta\sin p \\ \sin\theta\cos p - i\cos\theta\sin p \end{pmatrix}, \tag{2}$$

and

$$\mathbf{A} = \begin{pmatrix} -\sin(\theta+\Upsilon)\cos q - i\cos(\theta+\Upsilon)\sin q \\ \cos(\theta+\Upsilon)\cos q - i\sin(\theta+\Upsilon)\sin q \end{pmatrix}, \tag{3}$$

respectively, and the Jones matrix $\mathbf{M_H}$ is described as

$$\mathbf{M_H} = \begin{pmatrix} \cosh Qd + (ig_0 + p_0)\frac{\sinh Qd}{Q} & -(\omega + i\delta)\frac{\sinh Qd}{Q} \\ (\omega + i\delta)\frac{\sinh Qd}{Q} & \cosh Qd - (ig_0 + p_0)\frac{\sinh Qd}{Q} \end{pmatrix}, \tag{4}$$

where

$$Q = \sqrt{(p_0 + ig_0)^2 - (\omega + i\delta)^2} \fallingdotseq p_0 + ig_0. \tag{5}$$

Here, g_0, p_0, ω and δ are respectively given by

$$g_0 = \frac{\lambda}{\pi}(n_s - n_f) = \frac{\lambda}{\pi} \cdot LB = \frac{\Delta}{2d}, \tag{6}$$

$$p_0 = \frac{\lambda}{\pi}(m_s - m_f) = \frac{\lambda}{\pi} \cdot LD = \frac{E}{2d}, \tag{7}$$

$$\omega = \frac{\lambda}{\pi}(n_L - n_R) = \frac{\lambda}{\pi} \cdot CB = \frac{\Delta \cdot (-k)}{d} = ORP, \tag{8}$$

$$\delta = \frac{\lambda}{\pi}(m_L - m_R) = \frac{\lambda}{\pi} \cdot CD = \frac{\Delta \cdot k'}{d}, \tag{9}$$

where n and m denote the refractive indices and absorption coefficients, respectively, s and f represent slow and fast light rays, respectively, and L and R represent left and right-circularly polarized light, respectively; Δ and E represent the total phase difference and total linear dichroism (LD), respectively; d is the thickness of S, $k = -\frac{\omega}{2g_0}$ and $k' = \frac{\delta}{2g_0}$.

In the extended HAUP method, the Jones vectors $\mathbf{P}$ and $\mathbf{A}$ and the Jones matrix $\mathbf{M_H}$ are substituted into Eq. (1). After several approximations and the above coordinate transformations, the relative intensity ratio Γ is obtained as follows:

$$\Gamma(\theta', \Upsilon') = I/I_0 = A''(\theta') + B''(\theta')\Upsilon' + C''\Upsilon'^2, \tag{10}$$

$$A''(\theta') = H''_{11} + H''_{12}\theta' + H''_{13}\theta'^2, \tag{11}$$

$$B''(\theta') = H''_{21} + H''_{22}\theta', \tag{12}$$

$$C'' = H''_{31}, \tag{13}$$

where

$$H''_{11} \rightarrow \text{a term independent of } \theta' \text{ and } \Upsilon', \tag{14}$$

$$H''_{12} = 0, \tag{15}$$

$$H''_{13} = e^{E} + e^{-E} - 2\cos\Delta, \tag{16}$$

$$H''_{21} = -b'_1 p + b'_2 q + a_1 \delta\Upsilon - 2c_2(\sin\Delta)k, \tag{17}$$

$$H''_{22} = 2(e^E - \cos\Delta), \tag{18}$$

$$H''_{31} = e^E, \tag{19}$$

with

$$a_1 = \frac{2\sin^2\Delta}{e^E + e^{-E} - 2\cos\Delta}, \tag{20}$$

$$b'_1 = \frac{2(\cos\Delta - e^{-E})\sin\Delta}{e^E + e^{-E} - 2\cos\Delta}, \tag{21}$$

$$b'_2 = \frac{2(e^E - \cos\Delta)}{e^E + e^{-E} - 2\cos\Delta}, \tag{22}$$

$$c_2 = \frac{1}{K^2+1} = \frac{1}{(E/\Delta)^2+1}, \tag{23}$$

The extinction position angle θ_0 is given by

$$\theta_0 = -a_2(p+q) - b_2\delta\Upsilon + c_1 k + c_2 k' + N, \tag{24}$$

where

$$a_2 = \frac{\sin\Delta}{e^E + e^{-E} - 2\cos\Delta}, \tag{25}$$

$$b_2 = \frac{e^E - \cos\Delta}{e^E + e^{-E} - 2\cos\Delta}, \tag{26}$$

$$c_1 = \frac{K}{K^2+1} = \frac{E/\Delta}{(E/\Delta)^2+1}. \tag{27}$$

Here, N represents the absolute position of θ_0 (a meaningless value).

In the extended HAUP method, the values of LB, LD, ORP and CD are determined by the following procedure. First, the transmitted light intensities I are measured as double functions of θ' and Υ'. We measured transmitted light intensities of 90 discrete azimuthal orientations points for each wavelength. Next, the values of $I_0 H''_{ij}$ $(i, j = 1, 2, 3)$ and the extinction position angle θ_0 are determined by least-squares fitting using Eqs. (10)-(13). The

values of Δ, E and I_0 are calculated from the values of H''_{13}, H''_{22} and H''_{31}, Eqs. (16), (18) and (19). The LB and LD are obtained from Eqs. (6) and (7) and the sample thickness d. After evaluating the systematic error parameters q and $\delta\Upsilon$, the ORP and CD are obtained from the values of H''_{21} and θ_0 by least-squares fitting using Eqs. (17) and (24). In measurements on achiral (optically inactive) crystals such as MgF_2 and $LiNbO_3$, p (which is independent of the sample and the sample setting) was predetermined as 3.5×10^{-4}.[S8] The values of k and k' are then calculated by eliminating the systematic error parameters p, q, and $\delta\Upsilon$ from Eqs. (17) and (24). Finally, the ORP and CD are obtained by Eqs. (8) and (9), respectively.

In this study, we introduced Nd-Fe-B magnets to the G-HAUP, which apply a magnetic field parallel/anti-parallel to the light propagation direction. In this configuration, we could obtain the Faraday rotation (FR) and Magnetic-CD (MCD) in addtion to the ORP and CD. The origins of ORP and CD differ from those of FR and MCD, but the optical phenomena of all four parameters are the same,[S9] Therefore, when measuring FR and MCD, we don not need to modify the measurement principle of the G-HAUP.

Data analysis of the G-HAUP *a*-axis measurements

We prepared a 57.9-μm thick sample of the (100) plates of single-crystal CeF_3. The optical characters, *i.e.*, the directions of the fast and slow light rays, were determined under a polarizing microscope (DMLP, Leica, Hesse, Germany) with a Berek compensator. The absolute phase difference Δ is given by

$$\text{Absolute } \Delta = \pm \text{ as-measured } \Delta + 2n\pi (n \in \text{ Integer}). \tag{28}$$

The sign of as measured Δ and the index n were also determined using the Berek compensator.

Figure S2 shows the wavelength dependences of the absolute Δ, E, H''_{21} and θ_0 without the magnetic field application. From the H''_{21} and the θ_0 plots over the whole wavelength

region (excluding the unstable region 325 - 315 nm and the low-sensitivity region 445 - 400 nm), the systematic error parameters q and $\delta\Upsilon$ were determined by least-squares fitting as $q = -5.2 \times 10^{-4}$ and $\delta\Upsilon = -3.8 \times 10^{-4}$, respectively. Then $p = 3.5 \times 10^{-4}$ and q and $\delta\Upsilon$ were eliminated from the values of Δ, E, H''_{21} and θ_0. The LB, LD, OR and CD were successfully extracted and their wavelength dependences are shown in Figure S3.

Here, we can also find the anomalous ORD behavior near the unstable and low-sensitivity wavelength regions. In the G-HAUP measurement principle, the values H''_{21} and θ_0 include terms related to ORP and CD, depend on $1/2\cos\Delta$ and $\sin\Delta$. When the absolute value of Δ, (*i.e.*, total phase difference) is close to an integer multiple of $2n\pi (n \in$ Integer), the terms related to $\sin\Delta$ approach zero, so the the values of H''_{21} and θ_0 become unstable. Similarly, when the absolute value of Δ is close to an integer multiple of $(2n+1)\pi (n \in$ Integer), terms related to $1/2\cos\Delta$ approach ∞, diminishing the sensitivity of H''_{21} and θ_0. This instability is considered to underlie the anomalous ORD behavior. To avoid misunderstanding and misrepresentation, we must ignore data points within the unstable and low-sensitivity regions. In Figure S3 (c) and (d), the omitted data points are indicated in black. These data were eliminated in Figure 2 (c) and (d) of the main text.

We also measured the wavelength dependences of the absolute Δ, E, H''_{21} and θ_0 under an magnetic field applied parallel and anti-parallel to the light propagation direction. The results are shown in Figure S4 and S5, respectively. The systematic error parameters q and $\delta\Upsilon$ were determined as $q = -5.2 \times 10^{-4}$, $\delta\Upsilon = -2.1 \times 10^{-4}$ respectively in the parallel case and as $q - 4.2 \times 10^{-4}$, $\delta\Upsilon = -4.3 \times 10^{-4}$ respectively in the anti-parallel case. The wavelength dependences of the LB, LD, FR, and MCD in the parallel and anti-parallel cases are shown in Figure S6 and S7, respectively. In general, reversing the magnetic field direction inverts the signs of FR and MCD. To obtain accurate spectra, we averaged the absolute magnitudes between the two measurement sets. The averages are plotted as the red rhombuses in Figure 2 (c) and (d) of the main text.

References

(S1) Altomare, A.; Cascarano, G.; Giacovazzo, C.; Guagliardi, A. *J. Appl. Cryst.* **1993**, *26*, 343–350.

(S2) Sheldrick, G. M. *Acta. Cryst.* **2008**, *A64*, 112–122.

(S3) Cheetham, A. K.; Fender, B. E. F.; Fuess, H.; Wright, A. F. *Acta. Cryst.* **1976**, *B32*, 94–97.

(S4) Kobayashi, J.; Uesu, Y. *J. Appl. Cryst.* **1983**, *16*, 204–211.

(S5) Kobayashi, J.; Asahi, T.; Sakurai, M.; Takahashi, M.; Okubo, K.; Enomoto, Y. *Phys. Rev. B* **1996**, *53*, 11784–11795.

(S6) Tanaka, M.; Nakamura, N.; Koshima, H.; Asahi, T. *J. Phys. D: Appl. Phys.* **2012**, *45*, 175303–175310.

(S7) Kobayashi, J.; Kumomi, H.; Saito, K. *J. Appl. Cryst.* **1986**, *19*, 377–381.

(S8) Kobayashi, J.; Asahi, T.; Takahashi, S.; Glazer, A. M. *J. Appl. Cryst.* **1988**, *21*, 479–484.

(S9) Hecht, E. *Opics (5th Edition)*; Pearson, 2002.

Table S1: Crystal data, structure solution, and refinement details of single crystal CeF_3.

	CeF_3 single crystal
Chemical formula	CeF_3
Formula weight	197.12
Crystal color, habit	Colorless, block
Crystal dimensions	0.350 × 0.350 × 0.100 mm
Crystal system	Trigonal
Lattice parameters	a = 7.1277(6) Å<br>c = 7.2850(6) Å<br>V = 320.52(4) Å^3
Space group	P-3c1 (# 165)
Z value	6
Diffractometer	R-AXIS RAPID F (Rigaku, Tokyo, Japan)
Radiation	MoK$_\alpha$ (λ = 0.71075 Å)<br>graphite monochromated
Voltage, current	50 kV, 40 mA
Temperature	-100 °C
$2\theta_{max}$	54.8 °
No. of reflections measured	Total: 2829<br>Unique: 252 (R_{int} = 0.0769)
Structure solution	Direct methods (SIR92)
R_1 ($I > 2.00\ \sigma(I)$)	0.0736
R	0.0784
wR_2	0.1469
Goodness of fit	1.268
Max shift/error	0.000

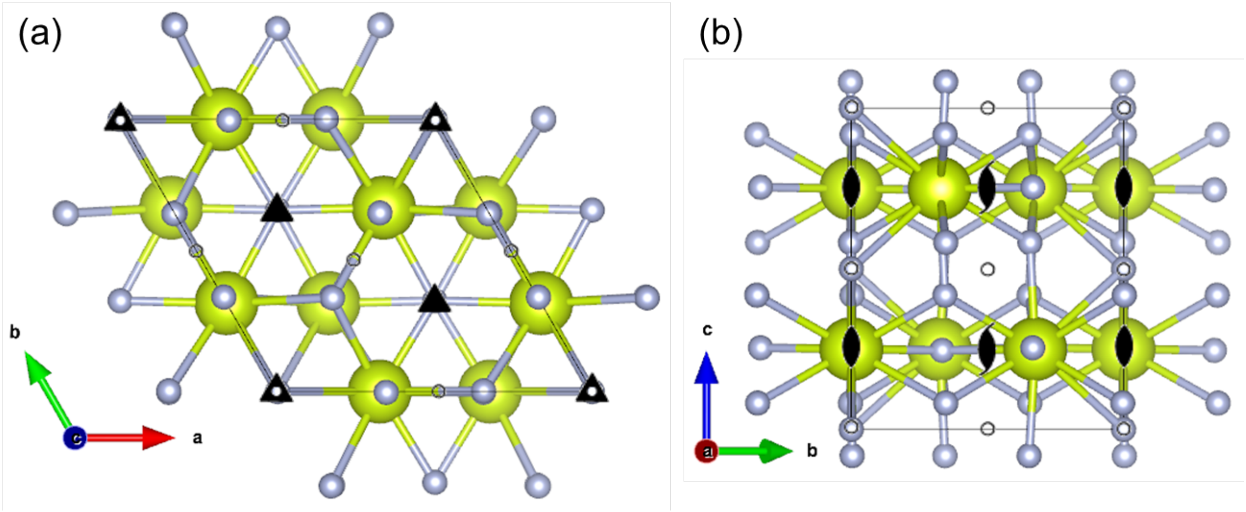

Figure S1: Crystal structure of a single crystal of CeF_3 viewed along the c axis (a) and a axis (b). The yellow and gray spheres represent Ce^{3+} ions and F^- ions, respectively.

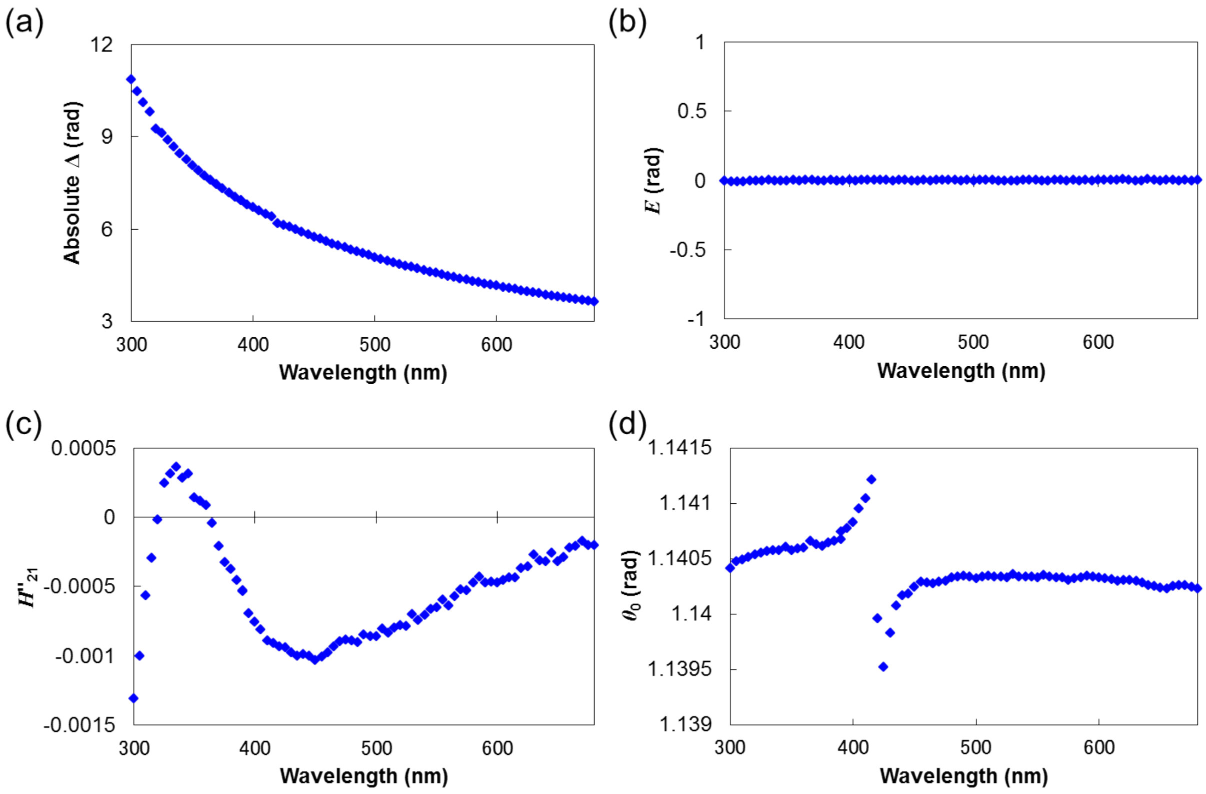

Figure S2: Wavelength dependences of the absolute Δ, E, H''_{21} and θ_0 without the magnetic field application.

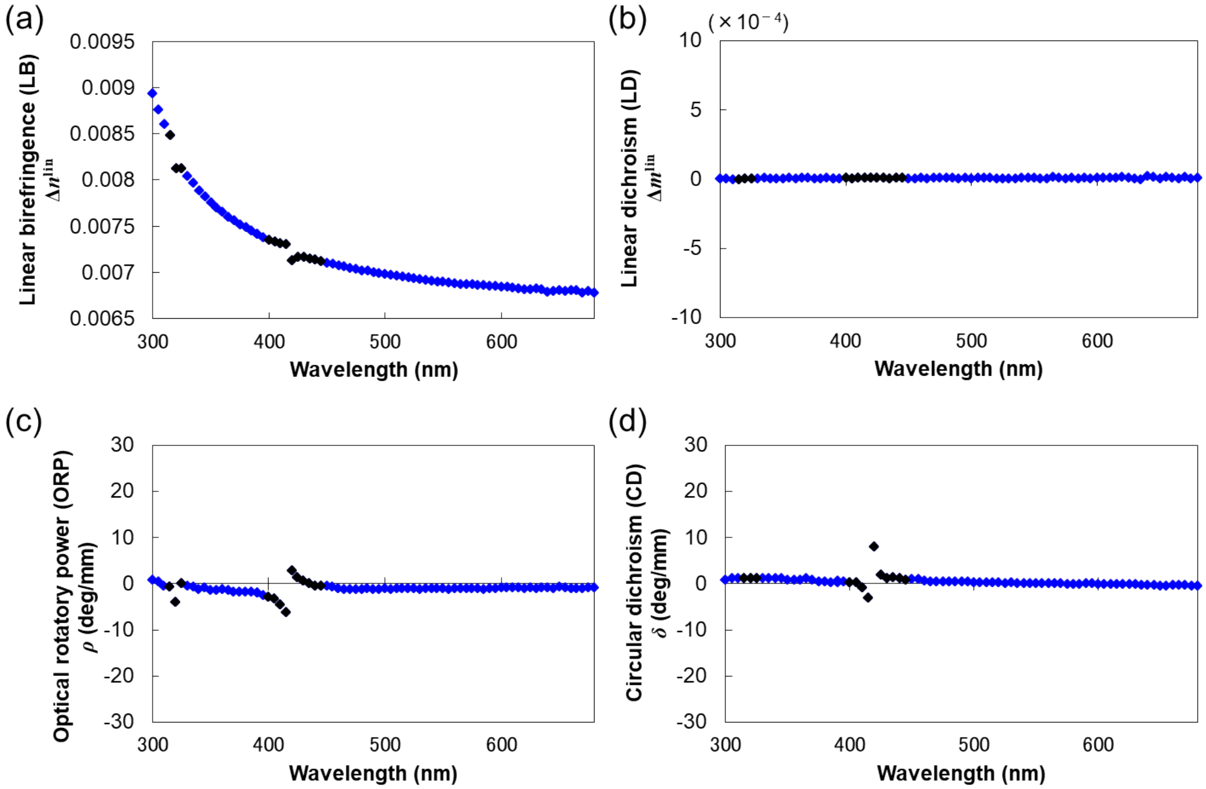

Figure S3: Wavelength dependences of the LB, LD, ORP and CD without the magnetic field application.

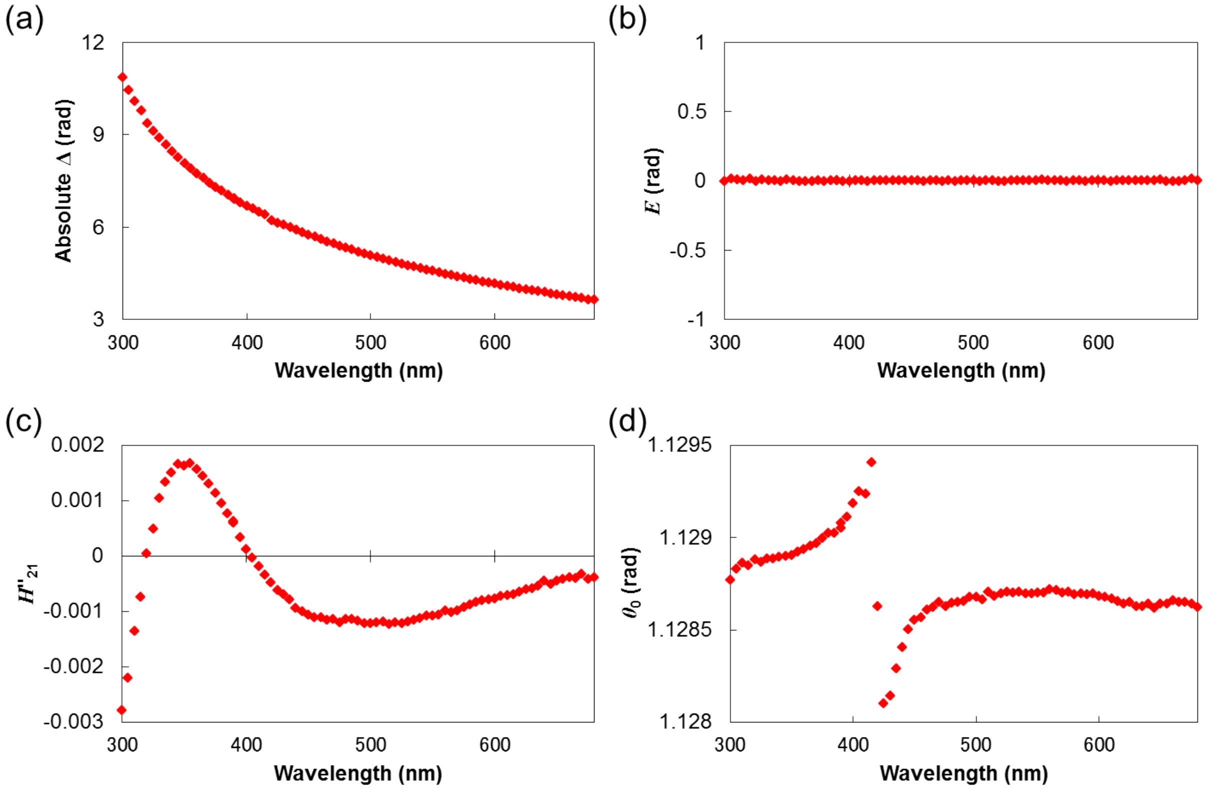

Figure S4: Wavelength dependences of the absolute Δ, E, H''_{21} and θ_0 under a magnetic field applied parallel to the light propagation direction.

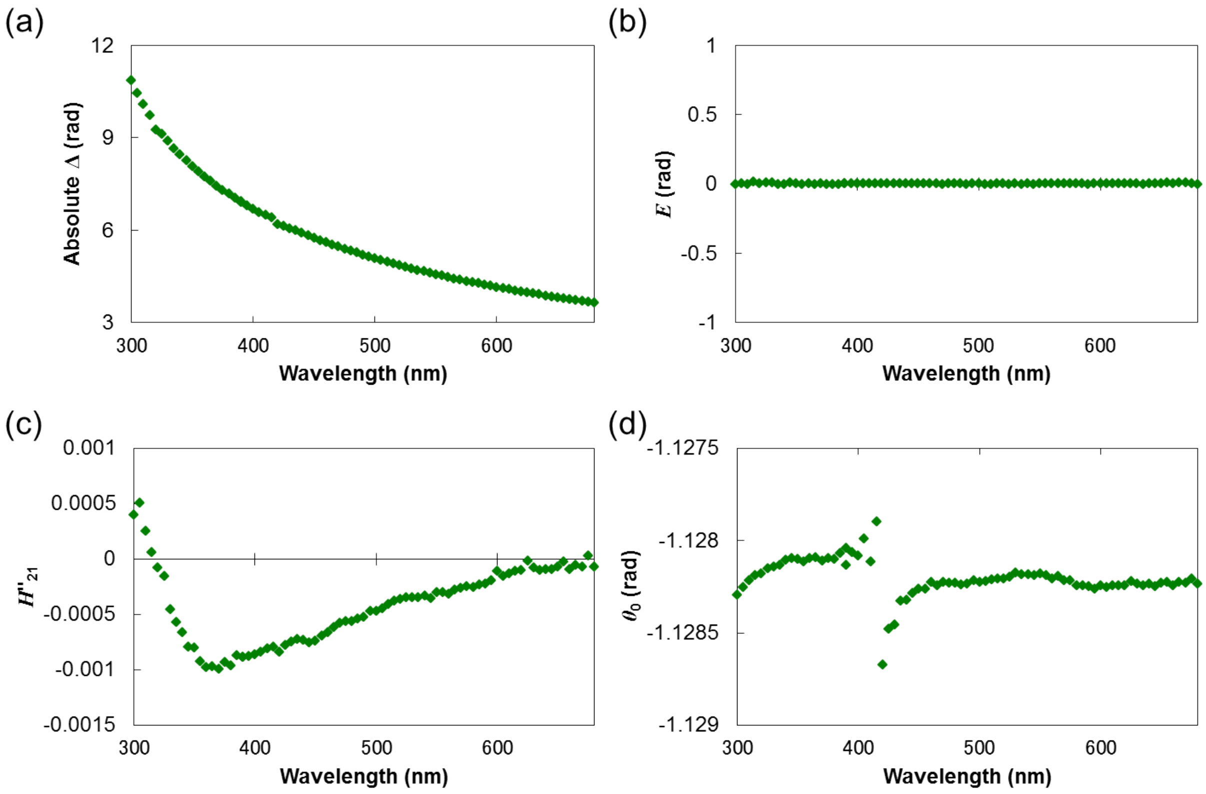

Figure S5: Wavelength dependences of the absolute Δ, E, H''_{21} and θ_0 under a magnetic field applied anti-parallel to the light propagation direction.

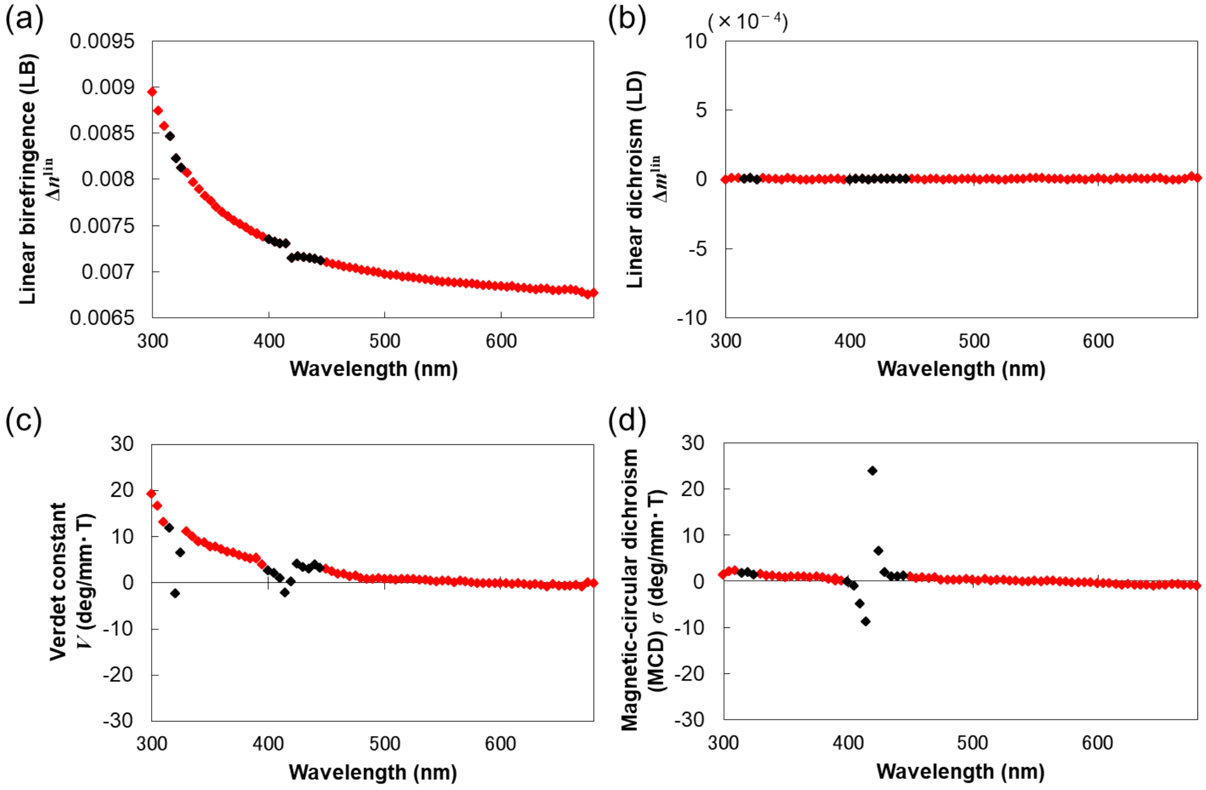

Figure S6: Wavelength dependences of the LB, LD, FR and MCD under a magnetic field applied parallel to the light propagation direction.

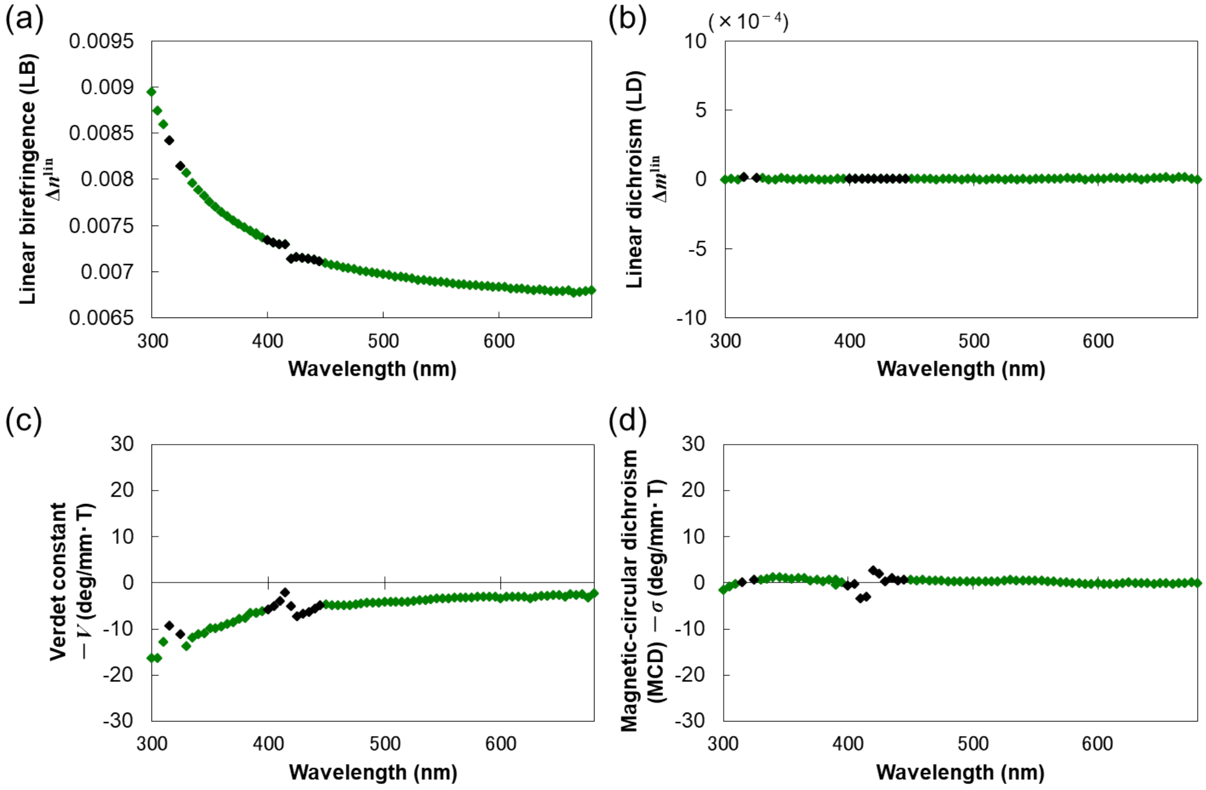

Figure S7: Wavelength dependences of the LB, LD, FR and MCD under a magnetic field applied anti-parallel to the light propagation direction.
